# Hepatic STAT1-Nuclear Translocation and Interleukin 28B Polymorphisms Predict Treatment Outcomes in Hepatitis C Virus Genotype 1-Infected Patients

**DOI:** 10.1371/journal.pone.0028617

**Published:** 2011-12-12

**Authors:** Tatsuo Miyamura, Tatsuo Kanda, Shingo Nakamoto, Shuang Wu, Keiichi Fujiwara, Fumio Imazeki, Osamu Yokosuka

**Affiliations:** Department of Medicine and Clinical Oncology, Graduate School of Medicine, Chiba University, Chiba, Japan; St. Louis University, United States of America

## Abstract

**Background:**

We investigated associations between signal transducer and activator of transcription (STAT) 1 in pretreated liver tissues, interleukin (IL) 28B polymorphism and treatment response in hepatitis C virus (HCV)-infected patients treated with peginterferon and ribavirin.

**Methods and Findings:**

We performed immunostaining analysis of STAT1 in liver tissues and determined IL28B polymorphism at rs8099917. We then compared the results with treatment outcomes in HCV genotype 1 patients with high viral load who were receiving peginterferon plus ribavirin. In univariate analysis, younger age, white blood cell counts, virological responder, early virological responder (EVR), mild activity (A1) of liver inflammation grading, and lower STAT1 nuclear-stain of hepatocytes in zone 1, zone 2 and total zones of liver were associated with sustained virological responder (SVR). Multivariate analysis showed that EVR, age and hepatic STAT1 nuclear-stain in zone 2 of liver were independent predictors of SVR. It was also revealed that IL28B and STAT1-nuclear translocation in hepatocytes are independent predictors of response to treatment with peginterferon and ribavirin in chronic hepatitis C patients.

**Conclusions:**

Concomitant assessment of lower STAT1 nuclear-stain of hepatocytes and IL28B polymorphism is useful for prediction of SVR in HCV genotype 1 patients.

## Introduction

Hepatitis C virus (HCV) chronically infects ∼170 million people worldwide and ∼3.1 million people in the US [1], [2]. Chronic HCV infection can cause chronic liver disease, cirrhosis and hepatocellular carcinoma (HCC) [3]. Treatment with peginterferon alfa and ribavirin leads to a sustained virological response (SVR) in ∼50% of patients infected with HCV genotype 1 with 48 weeks of therapy and ∼80% of patients infected with HCV genotype 2 or 3 with a 24-week course [4]–[6].

It has recently been reported that single nucleotide polymorphisms (SNPs) in the 19q13 region, in close proximity to three genes (IL28A, IL28B and IL29) encoding cytokines of the interferon lambda (i.e. type III interferon) family are strongly associated with the treatment response to peginterferon alfa and ribavirin among HCV genotype 1-infected individuals [7]–[10]. One of these SNPs, rs8099917, is reportedly highly predictive of a favorable treatment response among patients infected with Japanese HCV genotype 1. Null responsiveness to interferon was used in the analysis that endorsed rs8099917 [8].

Interferon lambda utilizes a receptor complex different from interferon alfa, but both types of interferon induce signal transducer and activator of transcription 1 (STAT1) and STAT2, as well as STAT3 activation [11]. Activation of the interferon receptor leads to at least one cytoplasm Janus tyrosine protein kinase (Jak1). Interferon stimulation results in tyrosine phosphorylation, dimerization, and nuclear import of STATs [12]. STATs and the interferon-stimulated gene factor 3 (ISGF3) transcription factor complex moves into the nucleus, binds to interferon-stimulated response elements (ISRE) in the promoters of the interferon-stimulated genes (ISGs) like 2′, 5′-oligoadenylate synthetase (OAS) and Myxovirus resistance protein A (MxA) genes, and induces transcription of those genes. Gene expression array analysis showed that interferons alfa and lambda induced a similar subset of genes although interferon lambda signaling was observed for more restricted cell lines. Interferon lambda has been shown to be induced after stimulation with several single-stranded RNA (ssRNA) viruses [13]. There was a report that the antiviral activity of type III interferon surpassed that of type I interferon [14]. There are several reports concerning HCV interfering with the Jak/STAT signaling pathway [15], [16].

As shown previously, nonresponders had high expression levels of ISGs before therapy [17]. Sarasin-Filipowicz et al. [18] reported that phospholyration, DNA binding, and nuclear localization of STAT1 were pre-activated and refractory to further stimulation in nonresponsive patients. Several recent studies suggested that the expressions of ISGs in liver [19] and plasma [20] are associated with genetic variation in IL28B and the outcome of interferon therapy for chronic hepatitis C. The expression of hepatic ISGs is strongly associated with treatment response and genetic variation of IL28B [19]. The favorable IL28B SNP variants are also associated with lower baseline interferon-gamma-inducible protein 10 kDa (IP-10 or CXCL10) [20].

The impact of STAT1 on the elimination of HCV RNA during therapy in the setting of IL28B genetic variants is unknown. We therefore assessed the nuclear translocation of STAT1 in pre-treatment liver biopsies and IL28B rs8099917 in patients chronically infected with HCV genotype 1. We also correlated the biochemical data with the treatment response, and all resulting data were analyzed.

## Materials and Methods

### Patients

Between February 2010 and June 2011, 202 patients with chronic hepatitis C were recruited into the present study at the Department of Gastroenterology, Chiba University Medical School Hospital, Chiba, Japan. Some of these patients had already been included in previous reports [10]. The baseline characteristics are listed in Table 1.

**Table 1:** Basic characteristics of patients infected with HCV.

IL28B rs8099917	Total	Major	Minor	*P*-value
No. of patients	202	134	68	
Age, y	55.4±11.7	56.6±11.1	53.1±12.4	0.0431
Gender (M/F)	101/101	64/70	37/31	NS
HCV RNA (H/L/U)	191/10/1	125/9/0	66/1/1	NS
Genotype (G1/G2/U)	166/34/2	111/22/1	55/12/1	NS
ASL (IU/L)	57.2±43.9	56.4±47.9	58.7±34.9	NS
ALT (IU/L)	69.9±60.0	67.5±63.3	74.7±53.2	NS
G-GTP (IU/L)	52.5±60.6	42.5±38.3	72.0±86.6	0.000949
WBC (/mm^3^)	5,240±1,520	5,270±1,620	5,170±1,320	NS
Hb (g/dL)	14.0±1.3	14.0±1.2	14.1±1.3	NS
Platelets (×10^4^/mm^3^)	17.3±5.8	17.5±5.7	17.1±6.0	NS
DM (+/−/U)	30/169/3	18/114/2	12/55/1	NS
US (CH/LC/U)	163/33/6	112/19/3	51/14/3	NS

We defined IL28B rs8099917 TT (n = 134) as major type and TG (n = 64) and GG (n = 4) as minor type.

H, high viral load (±5 log IU/mL); L, low viral load (<5 log IU/mL); G1, genotype 1; G2, genotype 2; U, unknown; WBC, white blood cell count; Hb, hemoglobin; DM, diabetes mellitus; US, ultrasound finding; CH, chronic hepatitis; LC, liver cirrhosis; NS, not significant.

All patients were adults, had compensated liver disease, and fulfilled the inclusion criterion of a positive test for anti-HCV antibody and HCV RNA. All these patients had samples available for IL28B analysis, and 79 of them underwent liver biopsy for samples for evaluation of hepatic STAT1. This study was approved by the ethics committee of Chiba University, Japan (permission number 247 and 840), and conformed to the Helsinki Declaration. Written informed consent was obtained from all patients before enrollment in this study. This work was partly presented at the HCV2011 18^th^ International Symposium on Hepatitis C Virus and Related Viruses on September 9, 2011, Seattle, WA, USA.

### Treatment

One hundred twenty-four patients were treated with pegylated interferon alfa once weekly and 400–1,000 mg of ribavirin orally twice daily [10]. Some of the patients stopped treatment at 12–16 weeks according to the early stopping rule. Fifteen, 78, and 28 received treatment for <48, 48 and 72 weeks, respectively. The duration of treatment was unknown in 3 patients. Seventy-nine of these 124 HCV patients, evaluated for hepatic STAT1, received treatment for 48 weeks or longer.

### HCV Genotyping

HCV genotype was determined using the antibody serotyping assay of Tsukiyama-Kohara et al. [21]. In this assay, HCV serotypes 1 and 2 correspond to genotypes 1a/1b and 2a/2b, respectively, according to the classification of Simmonds et al. [22]. It was reported that in 84% patients, genotypes determined by this serological genotyping assay showed complete agreement with those determined by group-specific PCR, with none revealing a group opposite to that of the HCV genotype, and also that the detection rate of this assay was even higher than that of the PCR assay.

### HCV RNA Quantification

HCV RNA was determined by an Amplicor HCV monitor assay, version 2.0 (range: 0.5–850 KIU/mL) (Roche Diagnostics, Tokyo, Japan), Amplicor HCV assay (Roche) or COBAS TaqMan HCV test (Roche) (range: 1.2–7.8 log IU/mL). The detection limit of this qualitative assay is 50 IU/mL, corresponding to 1.7 log IU/mL by COBAS TaqMan PCR assay [23]. We defined HCV RNA≥5 log IU/mL and <5 log IU/mL as high and low viral titer of HCV RNA, respectively.

### Classification of Treatment Outcome

Patients were classified as having achieved RVR and EVR if HCV RNA was undetectable (<50 IU/mL) in serum at treatment week 4 and week 12, respectively, and as having SVR if HCV RNA was undetectable in serum 24 weeks after the completion of therapy.

### Liver Biopsies

Liver biopsies were obtained from 79 patients, and the samples were processed for both histological evaluation (hematoxylin and eosin) and staining for STAT1.

### STAT1 Immunohistochemistry

Liver sections were cut from paraffin blocks and captured on electrically charged slides. Sections were dewaxed in xylene and taken through a series of ethanol washes. Antigen retrieval was performed by autoclave pressure for 10 minutes at full pressure in 10 mmol/L sodium citrate buffer (pH 7.0) (Mitsubishi Chemical Medience, Tokyo, Japan). Endogenous peroxidase activity was blocked by incubation in 3% hydrogen peroxide for 30 minutes. Anti-Stat1 rabbit monoclonal antibody (42H3, Cell Signaling Technology, Boston, MA) was incubated at a dilution of 1∶200 overnight at 4°C, and immunodetection was done with an ImmPRESS anti-rabbit Ig reagent kit (Vector Laboratories, Burlingame, CA). Sections were counterstained with Meyer's hematoxylin solution (Wako Pure Chemical Industries, Osaka, Japan). Zone areas were defined according to Rappaport's classification [24] as follows: zone 1, acini centered on the portal triad; zone 3, the circulatory peripheries of acini (adjacent to terminal hepatic vein); zone 2, acini between zone 1 and zone 3. We counted 200 cells in each zone, and then recorded the percentage of cells with positive STAT1-nuclear translocation per total cell numbers.

### DNA extraction

Human genomic DNA was extracted from 1 mL of blood samples using a QIAamp DNA Blood Midi Kit (QIAGEN, Tokyo, Japan) and quantitated on UV visible spectrophotometer Ultrospec 3,000 (Pharmacia Biotech, Piscataway, NJ).

### IL28B genotyping

DNA samples from patients and controls were genotyped for the IL28B rs8099917 polymorphism with a simple assay based on restriction fragment length polymorphism methods [10] or TaqMan SNP genotyping assay (Applied Biosystems Inc, Foster City, CA) using the ABI Step One real-time PCR system, according to the manufacturer's recommended protocols. TaqMan probes and primers were designed and synthesized by Applied Biosystems. We analyzed IL28B rs8099917 TT as major type and TG and GG as minor type in the present study.

### Statistical analysis

Data were expressed as mean ± standard deviation (SD). Differences were evaluated by Student *t*-test, Chi-square test, or Fisher's exact test. P<0.05 was considered statistically significant. Variables with P<0.05 at univariate analysis were retained for multivariate logistic-regression analysis. For all tests, two-sided P-values were calculated and the results were considered statistically significant at P<0.05. Statistical analysis was performed using the Excelstatistics program for Windows, version 7 (SSRI, Tokyo, Japan) and DA Stats software (O. Nagata, Nifty Serve: PAF01644).

## Results

### IL28B rs8099917 in Japanese patients infected with HCV

Characteristics of the 202 patients in the present study are summarized in Table 1. IL28B rs8099917 TT, TG and GG were 134, 64 and 4 patients, respectively. Patients with IL28B rs8099917 major type were significantly older than those with IL28B rs8099917 minor type (Table 1). There were no differences in gender distribution, HCV RNA levels, HCV genotype, ALT, AST, white blood cell counts, hemoglobin, platelet counts, complication with diabetes mellitus and ultrasound findings between these two groups, but we noticed that gamma-GTP levels of IL28B rs8099917 minor type were higher than those of IL28B rs8099917 major type (Table 1).

### IL28B rs8099917 and treatment response

Among the total patients, we analyzed 79 HCV genotype 1-infected patients who were treated with peginterferon plus ribavirin and underwent liver biopsies (Table 2). IL28B rs8099917 TT, TG and GG were 53, 25 and 1 patients, respectively. This distribution was similar to that of the total 202 HCV-infected Japanese patients (*statistically not significant*).

**Table 2:** No correlation between STAT1-nuclear translocation and IL28B genotypes.

IL28B rs8099917	Total	Major	Minor	*P*-value
No. of patients	79	53	26	
Age, y	54.4±11.6	55.8±10.4	51.5±13.5	NS
Gender (M/F)	37/42	24/29	13/13	NS
ASL (IU/L)	58.0±51.5	60.0±59.6	54.0±29.2	NS
ALT (IU/L)	69.2±61.7	71.1±70.4	65.4±39.5	NS
G-GTP (IU/L)	40.8±25.4	35.6±24.0	51.4±25.3	0.00849
WBC (/mm^3^)	5,190±1,320	5,330±1,420	4,900±1,070	NS
Hb (g/dL)	14.4±1.2	14.3±1.2	14.5±1.2	NS
Platelets (×10^4^/mm^3^)	17.0±6.5	17.3±6.1	16.5±7.3	NS
DM (+/−/U)	12/67/0	6/47/0	6/20/0	NS
US (CH/LC/U)	63/15/1	42/11/0	21/4/1	NS
VR/Null Responder	51/28	39/14	12/14	0.0319
RVR (+/−)	2/77	1/52	1/25	NS
EVR (+/−)	28/51	24/29	4/22	0.0182
SVR (+/−)	29/50	24/29	5/21	0.0445
Liver Biopsy				
F (F1/F2/F3/F4)	29/25/15/10	20/16/10/7	9/9/5/3	NS
A (A1/A2/A3)	28/43/8	20/29/4	8/14/4	NS
Nuclear-STAT1 (%)				
Zone 1	9.14±8.11	8.65±7.67	10.1±9.03	NS
Zone 2	3.78±3.94	3.74±4.20	3.86±3.43	NS
Zone 3	7.41±7.07	6.90±6.76	8.44±7.71	NS
Total	6.57±5.29	6.28±5.58	7.16±4.69	NS

We defined IL28B rs8099917 TT (n = 134) as major type and TG (n = 64) and GG (n = 4) as minor type.

U, unknown; WBC, white blood cell count; Hb, hemoglobin; DM, diabetes mellitus; US, ultrasound findings; CH, chronic hepatitis; LC, liver cirrhosis; VR, virological responder; RVR, rapid virological responder; EVR, early virological responder; SVR, sustained virological responder; F, Staging of Fibrosis; A, Grading of Activity; nuclear-STAT1, STAT1-nuclear translocation; NS, not significant.

The proportions of virological responders (VRs) and early virological responders (EVRs) in the 53 patients with IL28B rs8099917 major type were higher than those in the 26 patients with IL28B rs8099917 minor type (Table 2). It was also confirmed that gamma-GTP levels of IL28B rs8099917 minor type were higher than those of IL28B rs8099917 major type in this population (Table 2).

### IL28B rs8099917, STAT1-nuclear translocation in hepatocytes and liver histology

In the present study, histological analysis of the pre-treatment liver samples revealed that there was no association between IL28B genotypes and staging of fibrosis nor association between IL28B genotypes and grading of inflammatory activity of the liver (Table 2).

Liver fibrosis was not correlated with STAT1-nuclear translocation in hepatocytes, but the activity of liver inflammation was correlated with STAT1-nuclear translocation in hepatocytes [mild activity (A1) vs. moderate activity (A2) and severe activity (A3): P = 0.0016 in zone 1; P = 0.00026 in zone 2; P = 0.0022 in zone 3; P = 0.00018 in all zones; data not shown]. Further, there was no association between STAT1-nuclear translocation in hepatocytes and IL28B rs8099917 (Table 2).

### Predictors of SVR

Table 3 shows the pre-treatment and treatment factors of HCV genotype 1-infected patients between the achieving and not achieving SVR groups. Univariate analysis showed that age, white blood cell counts, platelet counts, achieving virological response, achieving early virological response, and STAT1-nuclear translocation in zone 1, in zone 2, and in total number contributed to the achievement of SVR (Table 3). Factors significantly associated with SVR by univariate analysis were then subjected to multivariate logistic regression analysis. In HCV genotype 1-infected patients with higher viral load, EVR, age and STAT1-nuclear localization in zone 2 were independent predictors of SVR (P<0.0001, P = 0.011 and P = 0.032, respectively).

**Table 3:** Correlation between STAT1-nuclear translocation and SVR.

Treatment response	Total	SVR	Non-SVR	*P*-value
No. of patients	79	29	50	
Age, y	54.4±11.6	49.0±13.6	57.5±9.0	0.00128
Gender (M/F)	37/42	18/11	19/31	0.0668
ASL (IU/L)	58.0±51.5	59.3±77.7	57.3±27.9	NS
ALT (IU/L)	69.2±61.7	79.5±90.3	63.3±36.2	NS
G-GTP (IU/L)	40.8±25.4	36.3±30.0	43.4±22.3	NS
WBC (/mm^3^)	5,190±1,320	5,610±1,410	4,940±1,220	0.0292
Hb (g/dL)	14.4±1.2	14.6±1.2	14.2±1.2	NS
Platelets (×10^4^/mm^3^)	17.0±6.5	18.9±5.1	15.9±7.0	0.0472
DM (+/−)	12/67	3/26	9/41	NS
VR/Null Responder	51/28	29/0	22/28	0.00000182
RVR (+/−)	2/77	2/27	0/50	NS
EVR (+/−)	28/51	21/8	7/43	0.000000610
Liver Biopsy				
F (F1/F2/F3/F4)	29/25/15/10	13/11/4/1	13/11/4/1	NS
F (F1+F2/F3+F4)	54/25	24/5	30/20	0.0649
A (A1/A2/A3)	28/43/8	15/13/1	13/30/7	NS
A (A1/A2+A3)	28/51	15/14	13/37	0.0394
Nuclear-STAT1 (%)				
Zone 1	9.14±8.11	6.49±5.68	10.6±8.93	0.0288
Zone 2	3.78±3.94	2.51±3.31	4.52±4.12	0.0280
Zone 3	7.41±7.07	5.66±6.42	8.42±7.30	0.0948
Total	6.57±5.29	4.77±4.51	7.61±5.46	0.0203

WBC, white blood cell count; Hb, hemoglobin; DM, diabetes mellitus; US, ultrasound finding; CH, chronic hepatitis; LC, liver cirrhosis; VR, virological responder; RVR, rapid virological responder; EVR, early virological responder; SVR, sustained virological responder; F, Staging of Fibrosis; A, Grading of Activity; nuclear-STAT1, STAT1-nuclear translocation; NS, not significant.

**Figure 1:**
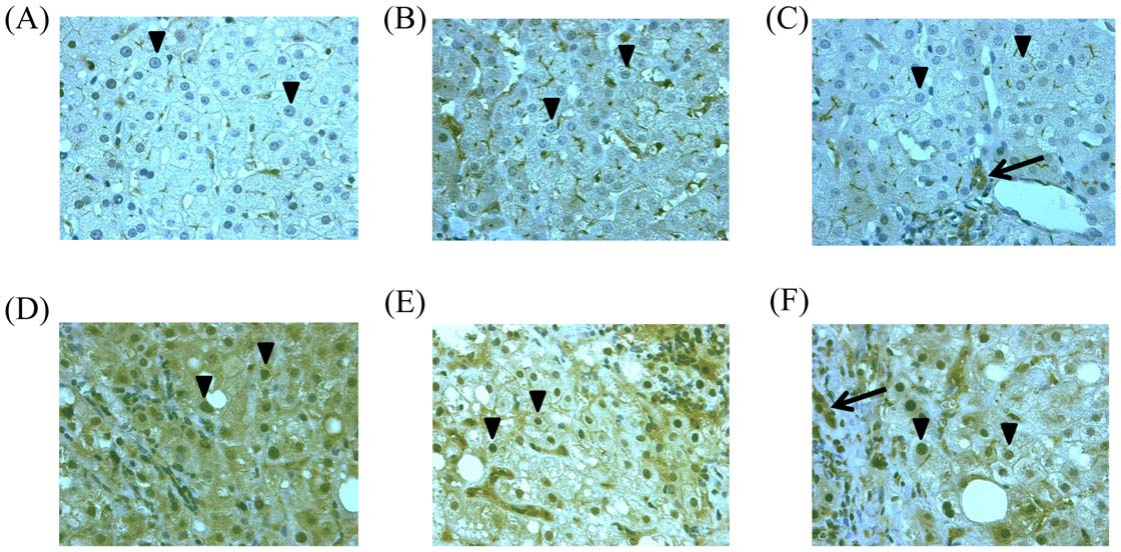
Immunohistochemistry for STAT1 in pretreated liver biopsy samples. (**A**)–(**C**) Patients with achievement of SVR. (**D**)–(**F**) Patients without achievement of SVR. (**A**) From a patient infected with HCV G1, high viral load (SVR, IL28B rs8099917TT). Arrowheads show markedly reduced nuclear staining for STAT1. (**B**) From a patient infected with HCV G1, high viral load (SVR, IL28B rs8099917TT). Arrowheads show markedly reduced nuclear staining for STAT1. (**C**) From a patient infected with HCV G1, high viral load (SVR, IL28B rs8099917TG). Arrow, bile duct; arrowheads show markedly reduced nuclear staining for STAT1. (**D**) From a patient infected with HCV G1, high viral load (Null responder, IL28B rs8099917TG). Arrowheads show more distinct nuclear staining for STAT1. (**E**) From a patient infected with HCV G1, high viral load (Relapser, IL28B rs8099917TT). Arrowheads show more distinct nuclear staining for STAT1. (**F**) From a patient infected with HCV G1, high viral load (Relapser, IL28B rs8099917TT). Arrow, bile duct; Arrowheads show more distinct nuclear staining for STAT1.

In the present study, only 2 of 79 HCV genotype 1-infected patients with high viral load achieved RVR, and these 2 patients also achieved SVR. Typical immune-histochemical analysis for STAT1 in hepatocytes is shown in Figure 1.

### Performance of IL28B rs8099917 and STAT1-nuclear translocation for predicting SVR

IL28B major genotype, which has been associated with better treatment response in several previous studies, was identified in 53 patients, and 24 of them (45.2%) achieved SVR (Tables 2 and 4). In the other 26 patients with IL28B minor genotype, 5 (19.2%) achieved SVR. STAT1-nuclear translocation in hepatocytes augmented the prediction of SVR among genotype 1-infected patients for IL28B major genotype (Table 4).

**Table 4:** Predictive values for SVR in patients infected with HCV genotype 1 (n = 79).

	PPV	NPV	Sensitivity	Specificity
**rs8099917 TT (n = 53)**	45.2%	80.7%	82.7%	42.0%
**Total nuclear-STAT1 (<5%) (n = 30)**	50.0%	71.4%	51.7%	70.0%
**rs8099917 TT or total nuclear-STAT1 (<5%) (n = 61)**	42.6%	83.3%	89.6%	30.0%
**rs8099917 TT and total nuclear-STAT1 (<5%) (n = 22)**	59.0%	71.9%	44.8%	82.0%

Nuclear-STAT1, STAT1-nuclear translocation; PPV, positive predictive value; NPV, negative predictive value.

## Discussion

In the present study, we confirmed that IL28B rs8099917 is useful for predicting null responders as was shown in previous studies. We also observed that STAT1, one of the ISGs, showed greater activation in pre-treatment hepatocytes of patients without SVR. Furthermore, the combination of STAT1-nuclear translocation in hepatocytes and IL28B rs8099917 favorable genotype is useful for the prediction of SVR in HCV genotype 1-infected patients with high viral load when they receive the combination peginterferon plus ribavirin therapy.

We observed the differences of STAT1-nuclear translocation in hepatocytes between SVR- and non-SVR-patients in zones 1 and 2, but not in zone 3. In chronic persistent hepatitis, weak membranous staining of human interferon gamma receptors was found on a number of scattered hepatocytes in acinar zone 1, with more pronounced expression on single hepatocytes in acinar zone 3 [25]. Honda et al. [26] reported that different interferon signaling in the liver lobule area (zone 2) and portal area (zone 1) under treatment for chronic hepatitis C. These data indicate that analysis of hepatocytes in each zone might be useful for the prediction of SVR in antiviral treatment of chronic hepatitis C patients.

In the case of West Nile virus (WNV) infection, only interferon lambda-induced activation of Jak-STAT signaling pathway and induction of ISG expression were completely inhibited in WNV replicon-containing cell lines, but interferon alfa signal transduction was either unaffected or only partially inhibited in Huh7.5 or Hela cells by the virus [27]. The differential inhibition of WNV by interferon alfa and interferon lambda signal transduction implies that there could be differences between the two types of interferon-receptors or -signalings. Further studies are also needed in HCV infection.

Rapid virological response (RVR) is well known to be an important predictor of SVR in combination therapy [6], [28]. However, RVR is less observed in HCV genotype 1-patients with high viral load. This means that other predictors are needed. IL28B variants were found not to be linked to STAT1-nuclear translocation (Table 2), although our present study had a rather small sample size. Our finding that STAT1-nuclear translocation was activated less in pre-treatment hepatocytes of patients with SVR than in those without SVR is in line with recent reports [19], [20]. There have been several reports that activation of ISGs at baseline levels of the liver among IL28B unfavorable carriers is typically observed in patients who respond less favorably to treatment [19], [29]–[31], although Dill et al. [32] reported that IL28B genotype and hepatic expression of ISG are independent predictors of response to treatment in chronic hepatitis C patients. Urban et al. [29] reported the fact that IL28B genotype may explain the relationship between hepatic ISG expression and HCV treatment outcome is independent of miR-122 expression. The present study is the first report concerning IL28B and STAT1-nuclear translocation in hepatocytes being independent predictors of response to treatment with peginterferon and ribavirin in chronic hepatitis C patients.

In conclusion, we found that IL28B and hepatic STAT1-nuclear translocation are independent predictors of treatment response. Concomitant assessment of pre-treatment STAT1-nuclear translocation in hepatocytes and IL28B rs8099917 favorable genotype improves the prediction of SVR in HCV genotype 1-infected patients with high viral load in the treatment of peginterferon plus ribavirin therapy.
